# Systemic CD3+ T-Cell Lymphoblastic Leukemia in a Bearded Dragon (*Pogona vitticeps)*: Clinical, Therapeutic, and Pathological Findings

**DOI:** 10.3390/ani15182736

**Published:** 2025-09-19

**Authors:** Josip Miljković, Anouk Jonker, Dražen Đuričić, Danijela Horvatek Tomić, Maja Belić, Siniša Faraguna, Mirela Pavić Vulinović, Ana Shek Vugrovečki, Maja Lukač, Ivan-Conrado Šoštarić-Zuckermann, Iva Šmit

**Affiliations:** 1 Department of Physiology and Radiobiology, Faculty of Veterinary Medicine, University of Zagreb, 10000 Zagreb, Croatia; ashek@vef.unizg.hr; 2 Department of Veterinary Pathology, Faculty of Veterinary Medicine, University of Zagreb, 10000 Zagreb, Croatia; anoukjonker@live.com (A.J.); isostaric@vef.unizg.hr (I.-C.Š.-Z.); 3 Department of Poultry Diseases with Clinic, Faculty of Veterinary Medicine, University of Zagreb, 10000 Zagreb, Croatia; ddjuricic@vef.unizg.hr (D.Đ.); horvatek@vef.unizg.hr (D.H.T.); mlukac@vef.unizg.hr (M.L.); 4 Department of Pathophysiology, Faculty of Veterinary Medicine, University of Zagreb, 10000 Zagreb, Croatia; mbelic@vef.unizg.hr (M.B.); sfaraguna@vef.unizg.hr (S.F.); 5 Department of Anatomy, Histology and Embryology, Faculty of Veterinary Medicine, University of Zagreb, 10000 Zagreb, Croatia; mpavic@vef.unizg.hr; 6 Internal Diseases Clinic, Faculty of Veterinary Medicine, University of Zagreb, 10000 Zagreb, Croatia; ismit@vef.unizg.hr

**Keywords:** bearded dragon, CD3 marker, chemotherapy, lymphoblastic lymphoma/leukemia, reptile oncology

## Abstract

A three-year-old male bearded dragon (*Pogona vitticeps*) exhibited acute anorexia. Biochemistry revealed mild hyperproteinemia (88 g/L) and elevated liver enzymes (ALT 60 U/L, AST 272 U/L), while the hematology report showed marked lymphocytosis. The animal had been clinically normal at a routine examination 10 months earlier. Based on the clinical and laboratory findings, acute lymphoblastic leukemia was suspected. Treatment was initiated with methylprednisolone (1 mg/kg PO q24h), marbofloxacin (10 mg/kg IM q24h), and lomustine (80 mg/m^2^ PO q14d), calculated according to reptile-specific body surface area formulas. A transient stabilization was followed by sudden deterioration on day 3, characterized by hematemesis and severe respiratory distress, leading to spontaneous death. A complete necropsy including histopathology and anti-CD3 immunohistochemistry revealed disseminated infiltration of neoplastic T-lymphocytes throughout all major visceral organs and confirmed the diagnosis of T-cell lymphoblastic lymphoma/leukemia (L/L). This case represents a rare report of systemic acute lymphoblastic L/L in a bearded dragon and underlies the importance of comprehensive diagnostics in reptiles with non-specific clinical signs and the challenges in the treatment of neoplastic diseases in exotic species.

## Simple Summary

The bearded dragon is a common pet reptile kept in numerous households around the world. This case report describes a three-year-old male bearded dragon that was brought to a veterinary clinic after it suddenly stopped eating. Although it was healthy at an examination ten months earlier, the animal showed signs of a significant pathological condition. Blood tests revealed abnormalities in the white blood cells and proteins that suggested leukemia, a type of cancer that affects lymphocytes, a subset of white blood cells. The animal was treated with medications, including corticosteroids, antibiotics, and an oral chemotherapeutic agent. Initially, the bearded dragon showed transient stabilization before rapidly deteriorating several days later. It developed breathing difficulties and vomited blood shortly before death. A necropsy (post-mortem examination) revealed that many internal organs, particularly the lungs, were severely infiltrated by abnormal lymphocytes. These findings confirmed the presence of an aggressive form of lymphoma/leukemia that had spread throughout the body. Lymphoblastic lymphoma/leukemia is a rare and severe disease and is difficult to treat in reptiles. This case highlights the importance of early diagnosis and the challenges of treating cancer in exotic animal species.

## 1. Introduction

Neoplastic diseases, once considered rare in reptiles, have been increasingly reported in recent studies, with a notable prevalence in lizard species [1,2]. Neoplasia is defined by the abnormal and uncontrolled proliferation of cells that persists regardless of the initiating stimulus and is not coordinated with the surrounding tissue structure [3]. In reptiles, the overall prevalence of neoplasia in lizards has been reported to range from 2.63% to 8.5%, with hematopoietic tumors accounting for a small but significant proportion [1,4]. Lymphoid malignancies, including lymphoblastic leukemia, chronic lymphocytic leukemia, and various forms of lymphoma, have been documented in bearded dragons [5,6,7,8,9,10].

Despite these reports, lymphoblastic lymphoma/leukemia (L/L) in reptiles remains poorly documented. There are only a few descriptions that include immunohistochemical confirmation or therapy trials in reptiles. Lymphoblastic leukemia is a clonal hematopoietic malignancy characterized by the proliferation of neoplastic B- or T-lymphocytes from the bone marrow, often involving peripheral blood and other tissues. In mammals, it is classified according to cellular maturity (acute or chronic) and immunophenotype (B-cells or T-cells) [11]. In acute T-cell lymphoblastic leukemia, malignant transformation occurs at an early stage of T-cell development in the thymus, typically at the pro-T cell stage [12,13]. Common neoplastic proliferations, such as round cell tumors, have been reported less frequently in snakes (boa constrictor [14], Indian python [15], diamond python [16], Aruba Island rattlesnake [17]), chelonians (Chinese box turtle [18], diamondback terrapin [19]), and lizards (desert spiny lizard [20], green tree monitor [21], bearded dragon [5,6,7,8,9,10]) than in mammals (e.g., cats [22,23], dogs [24]) or other animals.

This case report describes the clinical presentation, diagnostic evaluation, therapeutic management, and post-mortem findings of disseminated T-cell lymphoblastic leukemia in a pet bearded dragon, a comprehensive approach that has only recently become more routinely implemented in reptile medicine.

## 2. Case Description

A 1.3-year-old male central bearded dragon, weighing 408.6 g at initial presentation, was kept in a well-equipped indoor terrarium with UVB lighting (Arcadia ProT5 UVB Kit–Dragon 14%, Arcadia Reptile, Mepal, Cambridgeshire, UK) with a thermal gradient of 31 to 40 °C. The food consisted of crickets, *Zophobas morio* larvae, leafy greens, blueberries, and lettuce. On the 10 February 2022, the animal was presented to the Clinic for Birds at the Faculty of Veterinary Medicine, University of Zagreb, Croatia, due to transient anorexia and behavioral changes, including increased time spent in cooler areas of the enclosure. Clinical examination and full-body radiographs revealed no significant abnormalities. The animal recovered after the first visit to the clinic.

The animal remained reportedly healthy until 30 December 2022, when it developed acute anorexia. According to the owner, the bearded dragon had stopped eating insects and was now completely dependent on assisted feeding, which was largely unsuccessful. Nevertheless, clinical examination showed that body condition and alertness were maintained. Subsequent radiographs also showed no abnormalities. Supportive therapy, including assisted feeding with critical care diet (3.5 mL PO of EmerAid Omnivore^®^, LafeberVet, Cornell, IL, USA), and fluid replacement with Ringer’s solution 15 mL/kg SC (B. Braun, Germany) was administered. The coprological examination revealed a high burden of oxyurid eggs (+++). According to Carpenter and Harms (2023) [25], the animal was treated once with fenbendazole 40 mg/kg PO (Panacur^®^ 10% oral solution, MSD Animal Health, Milton Keynes, UK).

**Figure 1:**
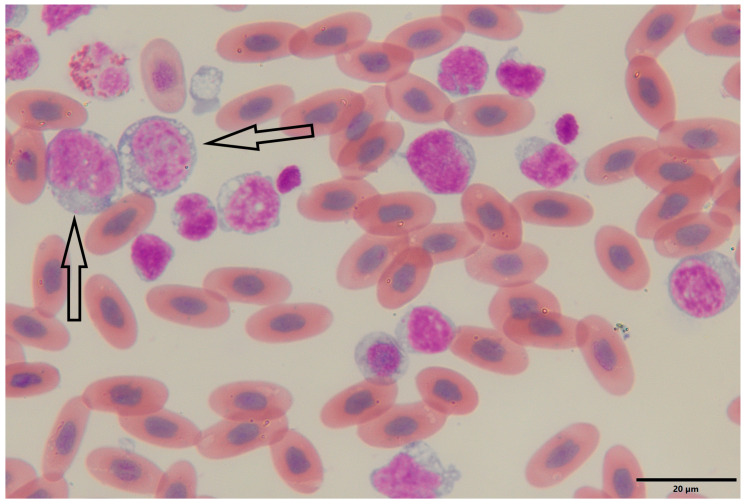
Peripheral blood smear of a bearded dragon (*Pogona vitticeps*) showing lymphoblasts. Wright–Giemsa-stained blood smear (oil immersion, 1000×) reveals a population of large, immature lymphoid cells (lymphoblasts) characterized by high nuclear-to-cytoplasmic ratios, round to oval nuclei with finely granular chromatin, and prominent nucleoli. The basophilic cytoplasm is variably vacuolated and frequently contains scattered granules (arrows). Mature erythrocytes are visible in the background as oval, nucleate, eosinophilic cells typical of reptilian blood. The abundance and morphology of the lymphoblasts are consistent with a diagnosis of lymphoblastic lymphoma/leukemia.

On the 26 January 2024, the bearded dragon was again anorexic. The animal was 3.3 years old and weighed 480 g at that time. The physical examination revealed a moderate body condition. Blood samples were collected for hematological and biochemical analysis in a 1.3 mL lithium heparin tube from the ventral tail vein. The hematological analysis showed an erythrocyte count of 0.9 × 10^6^/µL, a hematocrit of 38%, a hemoglobin concentration of 9.9 g/dL, MCV of 422.2 fL, MCH of 110 pg, and MCHC of 26.1 g/dL. Blood smear evaluation revealed a predominance of immature lymphoid cells with blast morphology, findings consistent with lymphoblastic L/L, although definitive classification requires immunophenotypic and clinical correlation (Figure 1). A total and differential leukocyte count could not be performed due to the overwhelming presence of the blast population. Biochemical analysis showed elevated liver enzymes (ALT 60 U/L, AST 272 U/L) and hyperproteinemia (88 g/L). The diagnosis of L/L was suspected, and immunocytochemical or immunophenotypic confirmation was recommended. Therapy was initiated with methylprednisolone 1 mg/kg PO q24h (2 mg tablets, Pfizer PFE GmbH, Berlin, Germany), marbofloxacin 10 mg/kg IM q24h for 10 days (20 mg/mL, Vetoquinol, Lure, France), fluid replacement with Ringer’s solution (B. Braun, Melsungen, Germany) and Duphalyte^®^ (Zoetis, Louvain-la-Neuve, Belgium) in a 4:1 ratio, 15 mL/kg SC, and 2 weeks later with lomustine 80 mg/m^2^ PO q14d (40 mg capsules, medac GmbH, Wedel, Germany) calculated according to published reptile-specific body surface area formulas [26].

**Figure 2:**
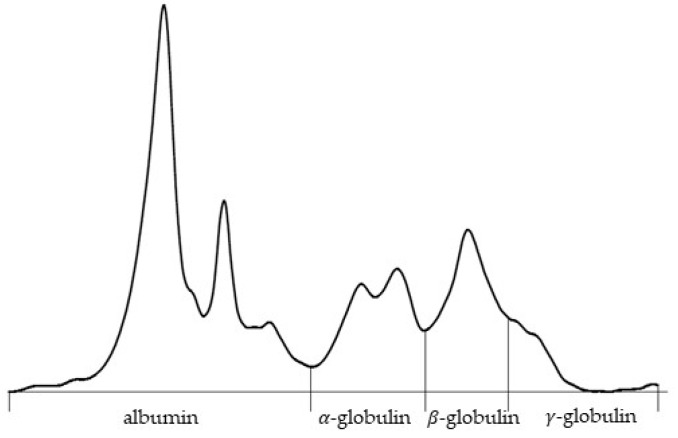
Serum protein electrophoresis curve of a bearded dragon (*Pogona vitticeps*). Quantitative analysis showed albumin (54.0%) and alpha-globulin (20.3%) fractions, with beta-globulin (19.2%) and gamma-globulin (6.5%) values. The total serum protein concentration was 34.8 g/L, resulting in an albumin-to-globulin (A/G) ratio of 1.17.

On the 5 February, the owner reported increased respiratory effort and mild ocular discharge. On the 7 February, the lizard’s dyspnea worsened, characterized by distension of the gular pouch during respiration, persistent anorexia, and obstipation. On the 10 February, the animal presented for administration of lomustine, marbofloxacin, and methylprednisolone. Despite persistent respiratory effort, the animal remained in good body condition and gained weight (486 g). Hematological analysis confirmed similar results as before (Table 1). Serum protein electrophoresis showed hyperalbuminemia (54%), increased alpha-globulins (20.3%), and decreased gamma-globulins (6.5%) (Figure 2). On the 13 February, a temporary improvement was observed, but the condition worsened acutely the next day. On the 14 February, darkening of the gular pouch and ventral body wall, accompanied by vomiting, diarrhea, and nervous behavior, was noted. The animal died spontaneously later that evening.

**Figure 3:**
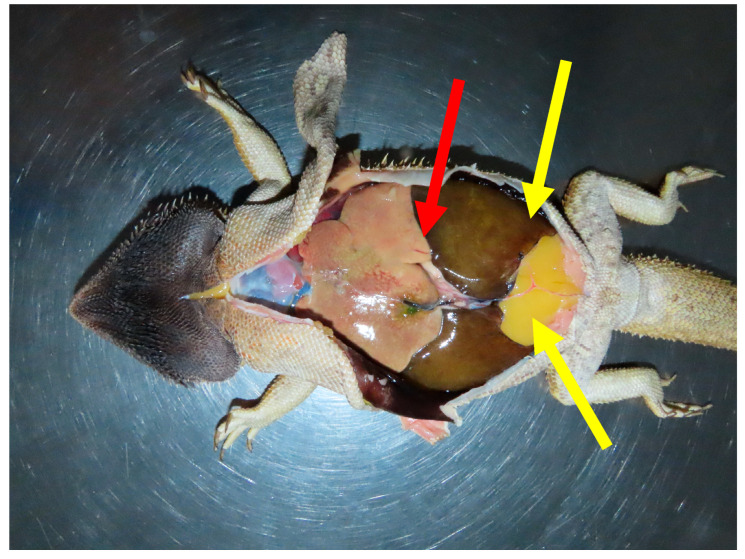
In situ post-mortem presentation of a bearded dragon (*Pogona vitticeps*), showing a markedly enlarged and pale liver (red arrow), prominent paired abdominal fat bodies (yellow arrows), and dark-brown to black discoloration of the skin of the ventral head.

A complete post-mortem examination was carried out within 24 h of death. The animal weighed 469 g and was in good nutritional condition (body condition score 6/9). Gross examination revealed dark discoloration of the gular skin and focal subcutaneous hemorrhage near the tail, likely related to recent venipuncture. The lungs were severely affected: the right lung was non-collapsing and filled with large volumes of clotted blood, while the left lung was partially collapsed and similarly contained clotted blood. The liver was significantly enlarged (32 g), diffusely pale, beige, with multifocal coalescing lighter yellow to white areas, and featured a focal, well-circumscribed white lesion on the capsule of the right lobe. The spleen was also markedly enlarged (33 × 12 mm), diffusely tan, and bulging on the cut surface. A small amount of blood was present in the oral cavity and stomach, whereas the intestines were largely empty or contained minimal mucoid content. All other organs appeared grossly unremarkable (Figure 3).

**Figure 4:**
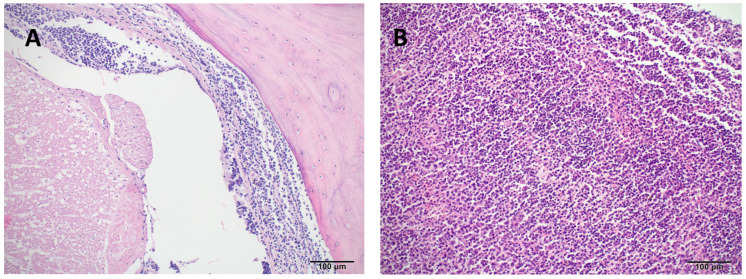
Hematoxylin and eosin (H&E) stained tissue sections of a bearded dragon (*Pogona vitticeps*) with systemic lymphoid neoplasia. (**A**) Cervical spine (10×): The meninges surrounding the spinal cord are extensively infiltrated and expanded by a dense population of neoplastic lymphoid cells. (**B**) Spleen (10×): Normal splenic architecture is diffusely effaced and replaced by a heterogeneous population of neoplastic lymphocytes.

Histopathological examination revealed widespread infiltration of neoplastic round cells in nearly all examined tissues, as specified later. In the liver, large portions of the parenchyma were replaced by these neoplastic cells, with concurrent macrovesicular lipid accumulation observed in the remaining hepatocytes. The spleen lacked normal follicular architecture, exhibiting diffuse infiltration by the same neoplastic population and a high number of apoptotic lymphoid cells. Neoplastic cell aggregates were also found in the heart, stomach, testis, kidney (including involvement of one glomerulus), small and large intestines, and extensively within the spinal dura and adjacent musculature (Figure 4). In the lungs, faveolar spaces and septa were multifocally filled with erythrocytes, proteinaceous fluid (edema), and neoplastic cells characterized by large nuclei and granular chromatin. Bone marrow evaluation was not possible due to artefactual distortion.

**Figure 5:**
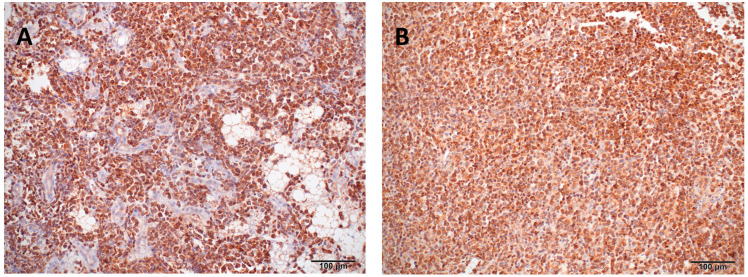
Immunohistochemical staining (CD3) of liver and spleen tissue from a bearded dragon (*Pogona vitticeps*). (**A**) Liver: The hepatic parenchyma is markedly disrupted and largely replaced by CD3-positive neoplastic lymphocytes. (**B**) Spleen: Diffuse infiltration of neoplastic lymphocytes exhibiting strong cytoplasmic CD3 immunopositivity, replacing the normal splenic architecture.

Immunohistochemistry on liver and spleen tissues revealed marked infiltration of CD3-positive neoplastic cells and effacement of tissue architecture. In mammals, the cytoplasmic staining with CD3 confirms a pro-T-cell lineage, as mature T-cell lineage expresses CD3 only in the cell membrane. Staining for B-cell markers CD20, CD79, and PAX5 was inconclusive or negative (Figure 5). These findings confirmed a disseminated T-cell lymphoproliferative neoplastic infiltration. The final pathological diagnosis included systemic infiltration by neoplastic CD3+ lymphoid cells, severe bilateral pulmonary hemorrhage, splenomegaly, and hepatic lipidosis. The presumed cause of death was acute respiratory failure secondary to pulmonary hemorrhage.

## 3. Discussion

Lymphoid neoplasms are increasingly recognized in reptiles, including bearded dragons, and are often associated with non-specific hematological abnormalities that may also indicate inflammation [27,28]. Although both leukemia and lymphoma have been reported sporadically in this species, their clinical course, histopathological features, and immunophenotypic profiles differ considerably. By definition, leukemia involves neoplastic round cells in the bone marrow or peripheral blood, whereas lymphoma is characterized by their presence in solid tissues; in cases where the primary site cannot be determined, as in the present case, the appropriate designation is lymphoma/leukemia. Various laboratory methods have been used to phenotype neoplastic hematopoietic cells and distinguish lymphomas from leukemias [1,3,4,5,10,29,30,31,32].

The present case describes a young male bearded dragon diagnosed with systemic T-cell acute lymphoblastic leukemia, which was confirmed by histopathological and immunohistochemical analysis. The clinical signs were non-specific—mainly anorexia and lethargy—but progressed rapidly and eventually ended in death. Laboratory testing revealed severe leukocytosis with a predominance of lymphoblasts and elevated liver enzyme activities, which are consistent with previous reports of leukemia in *P. vitticeps*, in which a high proportion of circulating immature lymphoid cells was similarly observed [5,31]. Immunohistochemical markers routinely employed in veterinary medicine were primarily developed and validated for use in mammals, with limited validation in reptilian tissues [4,22,23,32,33]. In the present case, strong cytoplasmic expression of CD3 was detected in infiltrative neoplastic cells, confirming T-cell lineage. B-cell neoplasia was excluded based on negative or inconclusive immunoreactivity for CD79a, CD20, and PAX5. This finding contrasts with a previously reported case of chronic lymphocytic leukemia in Pogona vitticeps, in which CD79a positivity indicated a B-cell origin [10]. Disseminated infiltration was observed in multiple organs, including the liver, spleen, kidneys, meninges, intestines, and lungs, leading to marked disruption of normal tissue architecture. Previous cases of leukemia in *Pogona vitticeps* described widespread infiltration without immunophenotyping, whereas the present case confirms a CD3+ T-cell neoplasm with notable meningeal and pulmonary involvement, highlighting underreported features [3,34].

Chemotherapy of L/L in reptiles is poorly documented. Due to species-specific metabolic and physiological differences, there are no standardised treatment protocols [35,36]. Treatment is largely derived from mammalian oncology, involving corticosteroids (e.g., prednisone) commonly used for their lympholytic effects and occasionally chemotherapeutic agents such as vincristine or cyclophosphamide [9,19,21,34,35,36,37,38,39,40]. Challenges include limited pharmacokinetic data, administration stress, and variable drug metabolism influenced by ectothermic physiology. Overall, therapy remains experimental, with supportive critical care for patient management.

A combination of lomustine, oral methylprednisolone, and marbofloxacin was used therapeutically in our study. This regimen was selected based on previous case reports indicating possible efficacy of lomustine and corticosteroids in reptiles with hematopoietic neoplasia [5,9]. In the study by Hepps Keeney et al. [5], partial clinical remission was achieved in a bearded dragon treated with lomustine and prednisolone, though relapse occurred within a few weeks. Reported adverse effects included heteropenia, gastrointestinal disturbances, and progressive systemic deterioration, particularly in advanced stages of disease. In this case, marbofloxacin was administered empirically; however, no evidence of bacterial infection was confirmed, and culture and sensitivity testing were not performed. We acknowledge that this represents a limitation of the therapeutic approach and emphasize that antimicrobial use in reptiles should be guided by diagnostic evidence and principles of antimicrobial stewardship.

In the present case, a slight transient clinical improvement was followed by rapid deterioration and death within a few days of initiating chemotherapy. Bilateral pulmonary hemorrhage, confirmed at necropsy, was considered a probable fatal event. The underlying cause of this decline remains unclear. It may be due to chemotherapy-induced toxicity, disease progression, or a combination of both. Possible mechanisms include tumor lysis syndrome—although rarely reported in reptiles, it remains a potential risk—along with vascular fragility due to leukemic infiltration or endothelial damage induced by lomustine. Prophylactic measures such as hydration were administered at each clinic visit. Given the extensive leukemic burden observed histologically, spontaneous lung failure due to leukostasis-like mechanisms is also a plausible explanation.

## 4. Conclusions

This case emphasizes several important points. First, cytology alone may be insufficient for the diagnosis of lymphoid neoplasms in reptiles; immunophenotyping is essential for accurate classification and prognosis. Secondly, although chemotherapy may provide a transient benefit, response to treatment is variable in reptiles, and pharmacokinetic data remain limited. Finally, the peracute terminal deterioration observed in this case highlights the limitations of current therapeutic protocols and emphasizes the need for further research into drug metabolism, dosing optimization, and supportive care in exotic species.

This case illustrates the diagnostic, therapeutic, and prognostic challenges associated with acute lymphoblastic leukemia in bearded dragons. The use of immunohistochemistry was essential to confirm the T-cell lineage of the lymphoblastic neoplasm and to distinguish it from previously reported B-cell leukemias. Despite the initiation of a chemotherapeutic protocol with lomustine and methylprednisolone, the clinical course was rapidly progressive and fatal, and the treatment was not completed, emphasizing the aggressive nature of the disease and the limitations of current treatment approaches in reptiles. Systemic infiltration, including lung and meningeal involvement, contributed to multi-organ dysfunction and sudden death. This report contributes to the growing literature on hematopoietic neoplasms in reptiles and highlights the urgent need for further research on diagnostic markers, pharmacokinetics of chemotherapeutic agents, and species-specific treatment protocols.

## Figures and Tables

**Table 1:** Hematological parameters of the bearded dragon compared with published reference intervals.

Data	RBC (×10^6^/µL)	HCT (%)	HGB (g/dL)	MCV (fL)	MCH (pg)	MCHC (g/dL)
26 January 2024	0.90	38	9.9	422.2	110.0	26.1
10 February 2024	0.95	28	9.63	294.7	101.4	34.4
Reference Interval [25]	0.4–1.6	17–45	4.7–14	77–506	16–163	19–46

## Data Availability

Supplementary data, including radiographic images, detailed hematology reports, high-resolution histopathology, and immunohistochemistry images, are available from the corresponding author upon reasonable request.

## Abbreviations

The following abbreviations are used in this manuscript:

L/L	Lymphoma/Leukemia
ALT	Alanine Aminotransferase
AST	Aspartate Aminotransferase
HCT	Hematocrit
HGB	Hemoglobin
MCV	Mean Corpuscular Volume
MCH	Mean Corpuscular Hemoglobin
MCHC	Mean Corpuscular Hemoglobin Concentration
RBC	Red Blood Cells
WBC	White Blood Cells
UVB	Ultraviolet B
IHC	Immunohistochemistry
CD3	Cluster of Differentiation 3 (T-cell marker)
CD20	Cluster of Differentiation 20 (B-cell marker)
CD79a	Cluster of Differentiation 79a (B-cell marker)
PAX5	Paired box gene 5 (B-cell marker)
H&E	Hematoxylin and Eosin (staining)
A/G	Albumin-to-Globulin Ratio
PO	Oral
IM	Intramuscular

## References

[1] Garner M.M., Hernandez-Divers S.M., Raymond J.T. (2004). Reptile neoplasia: A retrospective study of case submissions. Vet. Clin. N. Am. Exot. Anim. Pract..

[2] Kubiak M., Denk D., Stidworthy M.F. (2020). Retrospective review of neoplasms of captive lizards in the United Kingdom. Vet. Rec..

[3] Hernandez-Divers S.M., Garner M.M. (2003). Neoplasia of reptiles with an emphasis on lizards. Vet. Clin. N. Am. Exot. Anim. Pract..

[4] Solanes-Vilanova F., Hellebuyck T., Chiers K. (2025). Prevalence, diagnosis, treatment outcomes and immunohistochemical characterization of neoplastic disorders in reptiles presented at a veterinary teaching hospital: A cross-sectional study (2010–2023). Vet. J..

[5] Hepps Keeney C.M., Intile J.L., Sims C.S., Harrison T.M. (2021). Lymphoid leukemia in five bearded dragons (*Pogona vitticeps*). J. Am. Vet. Med. Assoc..

[6] Suedmeyer W.K., Turk J.R. (1996). Lymphoblastic leukemia in an inland bearded dragon. Bull. Assoc. Reptil. Amphib. Vet..

[7] Tocidlowski M.E., McNamara P.L., Wojcieszyn J.W. (2001). Myelogenous leukemia in a bearded dragon. J. Zoo Wildl. Med..

[8] Gregory C.R., Latimer K.S., Fontenot D.K., Lamberski N., Campagnoli R.P. (2004). Chronic monocytic leukemia in an inland bearded dragon, *Pogona vitticeps*. J. Herpetol. Med. Surg..

[9] Jankowski G., Sirninger J., Borne J., Nevarez J.G. (2011). Chemotherapeutic treatment for leukemia in a bearded dragon. J. Zoo Wildl. Med..

[10] Gavazza A., Galosi L., Croce V., Croce A., Genovese C., Romano P., Cerquetella M., Rossi G. (2019). A case of lymphocytic leukemia in a bearded dragon. Acta Vet. Beogr..

[11] Arber D.A., Orazi A., Hasserjian R., Thiele J., Borowitz M.J., Le Beau M.M., Bloomfield C.D., Cazzola M., Vardiman J.W. (2016). The 2016 revision to the World Health Organization classification of myeloid neoplasms and acute leukemia. Blood.

[12] Malcolm T.I., Hodson D.J., Macintyre E.A., Turner S.D. (2016). Challenging perspectives on lymphoma origins. Open Biol..

[13] Ruiz Pérez M., Vandenabeele P., Tougaard P. (2024). The thymus road to a T cell. Front. Immunol..

[14] Frye F.L., Carney J.D. (1973). Acute lymphatic leukemia in a boa constrictor. J. Am. Vet. Med. Assoc..

[15] Finnie E.P. (1972). Lymphoid leukosis in an Indian python. J. Pathol..

[16] Raiti P., Garner M.M., Wojcieszyn J. (2002). Leukemia and lymphoma in a diamond python. J. Herpetol. Med. Surg..

[17] Lock B., Heard D., Dunmore D., Ramaiah S. (2001). Lymphosarcoma with leukemia in a rattlesnake. J. Herpetol. Med. Surg..

[18] Bezjian M., Diep A.N., de Matos R., Schaefer D. (2013). Lymphoid leukemia in Chinese box turtle. Vet. Clin. Pathol..

[19] Silverstone A.M., Gamer M.M., Wojcieszyn J.W., Couto C.G., Raskin R.E. (2007). Acute leukemia in a diamondback terrapin, *Malaclemys terrapin*. J. Herpetol. Med. Surg..

[20] Goldberg S.R., Holshuh H.J. (1991). Leukemia in desert spiny lizard. J. Wildl. Dis..

[21] Georoff T.A., Stacy N.I., Newton A.N., McAloose D., Post G.S., Raskin R.E. (2009). Diagnosis and Treatment of Chronic T-Lymphocytic Leukemia in a Green Tree Monitor (*Varanus prasinus*). J. Herpetol. Med. Surg..

[22] Weyrich A., Hecht W., Köhler K., Herden C., Henrich M. (2024). Comparative analysis of primer sets for the assessment of clonality in feline lymphomas. Front. Vet. Sci..

[23] Siripoonsub J., Techangamsuwan S., Sirivisoot S., Radtanakatikanon A., Rungsipipat A. (2024). Investigating comparative polymerase chain reaction for antigen receptor rearrangement analysis in different types of feline lymphoma samples. Front. Vet. Sci..

[24] Fournel-Fleury C., Ponce F., Felman P., Blavier A., Bonnefont C., Chabanne L., Marchal T., Cadore J.L., Goy-Thollot I., Ledieu D. (2002). Canine T-cell lymphomas: A morphological, immunological, and clinical study of 46 new cases. Vet. Pathol..

[25] Carpenter J.W., Harms C.A. (2023). Reptiles. Exotic Animal Formulary.

[26] Keeney C.M.H., Nelson N.C., Harrison T.M. (2021). Use of computed tomography to determine a species-specific formula for body surface area in bearded dragons (*Pogona vitticeps*). Am. J. Vet. Res..

[27] Stacy N.I., Alleman A.R., Sayler K.A. (2011). Diagnostic hematology of reptiles. Clin. Lab. Med..

[28] Howard J.G., Jaensch S. (2021). Haematology and plasma biochemistry reference intervals in wild bearded dragons (*Pogona vitticeps*). Aust. Vet. J..

[29] Christman J., Devau M., Wilson-Robles H., Hoppes S., Rech R., Russell K.E., Heatley J.J. (2017). Oncology of reptiles: Diseases, diagnosis, and treatment. Vet. Clin. N. Am. Exot. Anim. Pract..

[30] Machotka S.V., Kaiser H.E. (1989). Lymphocytic neoplasms in reptiles and fish. Comparative Aspects of Tumor Development.

[31] Folland D.W., Johnston M.S., Thamm D.H., Reavill D. (2011). Diagnosis and management of lymphoma in a green iguana (*Iguana iguana*). J. Am. Vet. Med. Assoc..

[32] Rooney T., Ford A.K., Plattner B.L., Highland M.A., Eshar D. (2022). Pax5 and CD3 immunophenotyping of lymphoma in two central bearded dragons. J. Vet. Diagn. Investig..

[33] Gyimesi Z.S., Garner M.M., Burns R.B., Nichols D.K., Brannian R.E., Raymond J.T., Poonacha K.B., Kennedy M., Wojcieszyn J.W., Nordhausen R. (2005). High incidence of lymphoid neoplasia in a colony of Egyptian spiny-tailed lizards (*Uromastyx aegyptius*). J. Zoo Wildl. Med..

[34] Cannon M.J. (2003). Husbandry and veterinary aspects of the bearded dragon (*Pogona* spp.) in Australia. Semin. Avian Exot. Pet Med..

[35] Kent M.S. (2004). The use of chemotherapy in exotic animals. Vet. Clin. N. Am. Exot. Anim. Pract..

[36] Hahn K.A. (2005). Chemotherapy dose calculation and administration in exotic animal species. Semin. Avian Exot. Pet Med..

[37] Willig F., Torpy F.J., Harrison S.H., Duke E.G., Troan B., Boddy A.M., Abegglen L.M., Harrison T.M. (2024). Evaluation of neoplasia, treatments, and survival in lizard species. Animals.

[38] Zehnder A., Graham J., Antonissen G. (2018). Update on cancer treatment in exotics. Vet. Clin. N. Am. Exot. Anim. Pract..

[39] Dehghanpir S.D., Boudreaux B., Withers S.S., Izquierdo A., Sasaki E., Del Piero F., Braden M., Mitchell M.A. (2021). Chemotherapy-Responsive Acute Myeloid Leukemia in a Veiled Chameleon (*Chamaeleo calyptratus*). J. Herpetol. Med. Surg..

[40] Duke E.G., Harrison S.H., Moresco A., Trout T., Troan B.V., Garner M.M., Smith M., Smith S., Harrison T.M. (2022). A multi-institutional collaboration to understand neoplasia, treatment and survival of snakes. Animals.

